# The Sound of Silence: Mouse Models for Hearing Loss

**DOI:** 10.4061/2011/416450

**Published:** 2011-10-09

**Authors:** Sumantra Chatterjee, Thomas Lufkin

**Affiliations:** Stem Cell and Developmental Biology, Genome Institute of Singapore, 60 Biopolis Street, Singapore 138672

## Abstract

Sensorineural hearing loss is one of the most common disabilities in humans. It is estimated that about 278 million people worldwide have slight to extreme hearing loss in both ears, which results in an economic loss for the country and personal loss for the individual. It is thus critical to have a deeper understanding of the causes for hearing loss to better manage and treat the affected individuals. The mouse serves as an excellent model to study and recapitulate some of these phenotypes, identify new genes which cause deafness, and to study their roles *in vivo* and in detail. Mutant mice have been instrumental in elucidating the function and mechanisms of the inner ear. The development and morphogenesis of the inner ear from an ectodermal layer into distinct auditory and vestibular components depends on well-coordinated gene expression and well-orchestrated signaling cascades within the otic vesicle and interactions with surrounding layers of tissues. Any disruption in these pathways can lead to hearing impairment. This review takes a look at some of the genes and their corresponding mice mutants that have shed light on the mechanism governing hearing impairment (HI) in humans.

## 1. Introduction

The mammalian inner ear is a highly organized structure divided into auditory and vestibular components that are responsible for detecting and coordinating hearing, acceleration, and balance. The auditory organ consists of the coiled cochlea which senses sound. Inside this organ is a highly specialized epithelium that converts mechanical actions into electrical potentials. These epithelia contain sensory hair cells (HCs) as well as surrounding supporting cells. The hair cells are mechanoreceptors that can trigger action potentials in response to sound or movement. Damage to this small population of hair cells is a major cause of hearing loss.

There are two types of hearing impairment (HI): conductive and sensorineural. This classification is based on which part of the ear is affected. While conductive HI results from defects in the external or middle ear, sensorineural HI is due to malformations along the inner ear from the cochlea to the auditory cerebral cortex. A conductive defect generally leads to a less severe HI and can be solved with medical treatment. In contrast, a sensorineural defect leads to a range of HI, from less severe to extreme, and though sensorineurally hearing-impaired persons may be aided with cochlear implants or hearing aids, the problem is not completely solved [1].

In more than half of the patients with hereditary hearing loss (HHL), there are known genetic mutations. In most of these cases, a single gene is affected, resulting in the defect. About 70% of HHL cases in human are associated with a vestibular dysfunction only (nonsyndromic hearing loss (NSHL)) [2]. Various types of genes have been associated with HLL in humans. They include protein-coding genes (68) and tRNA- or rRNA- coding genes (7). (An extensive list can be found in the Hereditary Hearing Loss Homepage: http://hereditaryhearingloss.org/). The protein-coding genes primarily include transcription factors, ion channels and transporters, extracellular matrix components, gap junction and adhesion proteins, as well as myosins, cytoskeletal proteins, which all interact with each other to form a complex network that is critical for hearing.

## 2. One Stone Two Birds: Auditory and Vestibular Function of Inner Ear

To have a better understanding of the hearing loss, it is important to have a clearer picture of the formation of the inner ear. The mature inner ear, with its acoustic and vestibular components, is encased in the dense bone of the skull. Research over the years from multiple labs has helped us to have a better understanding of how the inner ear is shaped from a simple otocyst during embryonic development.

While the otocyst initially consists of simple pseudo-stratified epithelium, it soon undergoes extensive proliferation and differentiation that will eventually establish the ventrally derived auditory component, the cochlea, and the dorsally derived vestibular apparatus. In mammals, hearing is initially mediated through sensory cells located within the coiled cochlea. Almost all of the cell types within the membranous labyrinth of the inner ear are derived from multipotent epithelial progenitor cells initially located in the otocyst. Otocyst-derived cells develop into three major lineages, prosensory (cells that will develop as either hair cells or associated supporting cells), proneural (cells that will develop as auditory or vestibular neurons) and nonsensory (all other otocyst derived cells) with cells within each lineage developing in a precise spatiotemporal manner. Gene knockout studies have identified specific signaling molecules and pathways, including *Notch*, *Hedgehog*, *Sox2,* and *Fgfs*, that guide progenitor cells to develop first as a sensory precursor and subsequently as one of the more specialized cell types. For a detailed review on cochlear development, see [3, 4]. Highly differentiated sensory hair cells develop within the coiled cochlear duct to form the organ of Corti, which is responsible for detecting sound. Likewise, sensory hair cells arise within the vestibular apparatus to form the maculae in the utricle and saccule and the cristae in the semicircular ducts. Collectively, they are responsible for detecting gravity as well as maintaining balance [5, 6], for review see [7]. Generally, genes expressed in the ventral otocyst are implicated in the formation of auditory structures, while genes expressed in the dorsal otocyst are implicated in the formation of the vestibular apparatus.

*Pax2, *which is an early marker of otic fate, is subsequently required for cochlear development, and its inactivation in mice leads to cochlear agenesis [8]. *Pax2* is also expressed in the endolymphatic duct [9]. However, the vestibular apparatus and endolymphatic duct develop normally in the *Pax2* null mice, maybe due to compensation from other *Pax *genes, possibly *Pax8*. Detailed analysis of *Pax2/Pax8* double null mice could substantiate and clarify the roles of both genes during inner ear development.

In the *Otx1* null mice, both the lateral semicircular duct and the lateral sensory cristae are absent, similar to *Prx1/Prx2* double null mice, suggesting that *Otx* and *Prx* genes may interact with each other via other genes during inner ear development. The ventral-apical expression domain of *Otx2* in the otic epithelium gives rise to the saccule and a portion of the cochlea. Since *Otx2* null mice die prior to morphogenesis of the inner ear beyond the otic vesicle, *Otx2* function during inner ear development has been inferred by the analysis of *Otx1* null mice that are also heterozygous for *Otx2* [10–12]. The inner ear defects in these mice are more extensive than those reported for the *Otx1* null mice [10]. The defects to the cochlea are expanded ventrally and the saccule, which is unaffected in *Otx1* null mice, is dysmorphic. Therefore, *Otx2* expression appears to function both redundantly and independently of *Otx1* in establishing proper specification of the cochlea and saccule. Unlike *Otx1*, *Otx2* expression does not appear to be mediated by Shh signaling as ectopic expression of *Shh* in mice does not induce concomitant ectopic expression of *Otx2* [13]. 

*Gbx2 *expression eventually becomes restricted to the endolymphatic duct and ceases in the inner ear by E15.5 [14]. The knockout analysis showed a key role of *Gbx2 *in patterning of the dorsomedial regions (endolymphatic duct, vertical pouch) [14] leaving *Gbx2* null mice with a phenotype similar to that described for *kreisler* mice [15], mainly the absence of the endolymphatic duct and swelling of the membranous labyrinth. In the most severe cases, even the ventral inner ear structures were affected [14]. For a more detailed review of the inner ear development, see [16].

## 3. Of Mice and Men

The study of sensorineural HHL in humans is severely hampered by the absence of tools to study inner ear development *in vivo* as well as absence of cell lines, which recapitulate this process. Though genetic linkage analysis of HHL in humans is one possibility, it is limited by the requirement of a large family with many HI patients. Over the years, mouse has been a model system of choice for human diseases due to its evolutionary closeness. Hence, mutant mouse models that exhibit HHL may help to identify genes that have a role in the development or function of the inner ear. Many hearing-impaired mutant mice have arisen spontaneously during the last century. Also X-ray, chemical (by ENU, N-ethyl-N-nitrosourea and chlorambucil), gene trapped or targeted mutants have resulted in many new hearing-impaired mouse strains. Mutations in about 180 different genes have been reported as responsible for inner ear malformations in mice (http://hearingimpairment.jax.org/). After locating the affected gene, the mutant mice can be used for a spatiotemporal study of the gene, in an attempt to pinpoint the exact role of the gene in various pathways and processes involved in shaping the inner ear. But as with any model system, the mice do not and cannot recapitulate all the genes and pathways in human as only 44 of these genes have been linked to human HHL. In addition, two genes that were linked with human HHL were found as not crucial for inner ear development and function in knockout mice [2]. 

**Figure 1:**
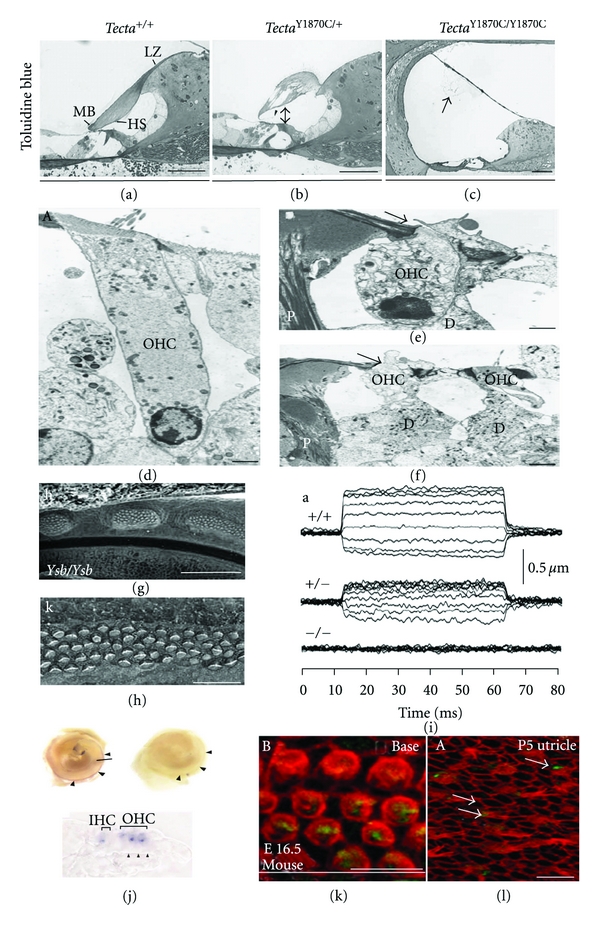
Assays to detect phenotypes of deafness-related mutations in mice. ((a)–(c)) Light microscopy analysis of sections of cochlear duct from (a) wild type, (b) *Tecta* ^Y1870c/+^, and (c) *Tecta* ^Y1870c/Y1870C^. LZ: limbal zone, and MB: marginal band, HS: Hensen's stripe. Arrowhead in (b) Kimura's membrane, arrow in (c) tectorial membrane. Reprinted with permission from [17]. ((d)–(f)) Transmission electron micrograph of the organ of Corti in wild type versus when Cx26 was deleted (e-f). D: deiters cell, P: outer pilla cells. Reprinted with permission from [18]. ((g)-(h)) Scanning electron micrograph of Organ of Corti basal portion at P0 from yellow submarine (Ysb) homozygous. Reprinted with permission from [19]. (i) In vitro analysis of OHC electromotility in wild type and mutant (Slc26a5) mice. It shows length changes of OHC in response to voltage steps (−120–60 mV in 20 mV steps) in whole-cell, voltage-clamp recordings. Reprinted with permission from [20]. (j) In situ hybridization detects expression of Cdh23 in the neurosensory epithelium of 4-day neonates. Antisense probe specifically labels the neuroepithelium along the cochlea duct (arrowheads). Cross-section through the cochlear duct identifies specific labeling in three outer (OHC) and one inner hair cells (IHCs). Reprinted with permission from [21]. (k) Immunohistochemistry on whole mount shows Cdh2 (green) is detected at E16.5 in stereocilia (stained for F-actin, red) of hair cells from the basal turn of the cochlea in wild-type mice. (l) Immunolocalization of cadherin 23 (shown in green) in the inner ear of homozygous waltzer v^6j^ mice. Cadherin 23 (green) was not detected in utricle stereocilia (stained for F-actin, red), but was detected in the cuticular plate of hair cells (arrows). Reprinted with permission from [22].

Over the years, the many assays used to evaluate the phenotype of deafness-related mutations have been available in the mouse model, making it an ideal system to study human deafness. These assays include, but are not limited to microcopy—bright field, transmission and scanning electron [17–19] (Figures 1(a)–1(h)), as well as by paint-fill analysis [23]. Physiological test, such as patch clamp assays, may be used to measure currents or membrane potentials in a single cell. Change in length of individual cells may be used to measure electromotility of outer hair cells [20] (Figure 1(i)). Temporal and spatial expression patterns of specific mRNAs can be observed by *in situ *hybridization in wild type and mutant inner ears [21, 24] (Figure 1(j)), and localization of various proteins can be detected by immunofluorescence [21, 22, 25, 26] (Figures 1(k)-1(l)). 

## 4. Mutant Mice for Genes of the Hair Bundle

Five genes have been implicated in NSHL in humans that directly have a role in either development and/or maintenance of the hair cell bundle. They are *MYOVIIA*, *USH1C/Harmonin*, *CDH23, PCDH15, *and *VLGR1/MASS.* These genes also contribute towards the progression of Usher syndrome, which is a combination of hereditary deafness and blindness. It is marked by sensorineural hearing loss and loss of visual field due to retinitis pigmentosa.

It has been shown that all the Usher-related proteins are bound to each other through the harmonin PDZ sites and form a multiprotein unit that can move along the hair cell actin filaments to their site of action within the stereocilia [27]. In the hair bundle, the stereocilia are arranged in rows with a special staircase pattern. The Usher-related proteins participate in interstereociliar links essential for mechanotransduction, a process where the cochlear and vestibular hair cells translate mechanical movements of their hair bundles to electrochemical signals. 

Cadherin 23 (*Cdh23*) and protocadherin 15 (*Pcdh15*) are transmembrane proteins with a short intracellular and a long extracellular domain. In their cytoplasmic end, *Cdh23* and *Pcdh1*5 contain class-I-PDZ-binding motifs (PBM) that can bind PDZ-containing proteins like Harmonin. In the wild-type mouse, *Cdh23* is localized to the hair cell stereocilia and Reissner's membrane [22, 28]. *Pcdh1*5 is widely expressed in multiple tissues, including the brain, cochlea, and vestibule [29]. In the developing cochlea, *Pcdh15* was localized to the apical surface of hair cells, supporting cells, outer sulcus cells, and spiral ganglion cells, while mature cochleae express protocadherin 15 only in hair cell stereocilia [30].

The first reported mutant for *Pcdh15 *was Ames-waltzer (*av*). Ames-waltzer mice were known to harbor a recessive spontaneous mutation causing deafness, circling behavior, head tossing, and hyperactivity, which were very similar to the waltzer (*v*) phenotype. Subsequently, many mutations in the same locus arose independently, resulting in similar phenotypes. The mutated gene was found to be *Pcdh15 *in an Ames-waltzer allele that was raised in transgenic mice following insertional mutagenesis [31]. Circling behavior and a reduced AM1-43 dye uptake, that correlates with normal transduction in hair cells, preceded structural defects in the vestibule that could be observed by light or scanning electron microscopy. While inner ears of P10 homozygotes displayed only abnormal stereocilia in the cochlea, by P30 the saccular stereocilia began to be affected. Adult homozygous mice had an almost complete degeneration of the organ of Corti and vestibular saccular macula. In the cochlea, degeneration of the spiral ganglion neurons was also observed. The neuroepithelia of the utricle and the semicircular canals cristae had a normal morphology, but the utricular otoconia were large and malformed.

There are four different spontaneous mutants for *Cdh23 *in mice: *waltzer* [21, 28], *waltzer niigata* [32], modifier of deafwaddler—*mdfw* [33], and age-related hearing loss—*Ahl* [34]. Chemical mutagenesis by chlorambucil—deletion mutations—(*Albany-waltzer* [35]) and ENU point mutations have also given rise to *Cdh23 *mutated mice. All but one (*Ahl*) of these-*Cdh23*-mutated mouse strains displayed a similar phenotype: NSHL with circling behavior, head tossing, and erratic movements that appear in homozygotes from birth. Heterozygotes appeared normal at birth, but developed a progressive hearing loss with aging and had a higher sensitivity for noise-induced hearing loss [36]. Waltzer mouse mutants also display a progressive disorganization of the hair bundle, which is first noted at the beginning of the bundle formation at E18.5.

The first spontaneous mouse mutant for *Vlgr1 *was *Mass*1^Frings^. This is used as a model for epilepsy due to its susceptibility to loud-noise-induced seizures. The BUB/BnJ inbred mouse strain is naturally homozygous for the *Mass*1^Frings^ mutation and is prone to sound-induced seizures and progressive hearing loss leading to complete deafness [37, 38]. Interestingly, BUB/BnJ mice are also homozygous for the *Ahl *allele of *Cdh23, *but unfortunately this is not enough to explain the deafness of these mice because other strains that are homozygous to* Ahl *do not display the severity of hearing loss. The association of *VLGR1 *mutations with HHL in Usher type II syndrome in humans [39] pointed to the fact that the *Mass*1^Frings^ mutation underlies hearing loss in BUB/BnJ mice. Indeed, it was shown that the double mutants of *Cdh23 *and *Vlgr1 *were responsible for most of the hearing loss in BUB/BnJ mice. In young BUB/BnJ mice, the cochlear stereocilia developed abnormally and remained in an underdeveloped state. Stereocilia were disconnected and detached, the most severely affected bundles lost their polarity and graded height. At older ages, hair cells and spiral ganglion cells were degenerated [40].

There are two mutant *Vlgr1 *mice, a knockout mouse that expresses no Vlgr1 proteins [41] and Vlgr1/del7TM mice, in which the transmembrane domain is deleted [42]. In both models, the absence of Vlgr1 receptors resulted in cochlear abnormalities. Homozygous mice did not display ankle links between the hair cell stereocilia. Although the hair bundles appeared normal at birth, they became disorganized thereafter. Homozygous mutant for *Vlgr1 *developed acute deafness by the third week of birth and henceforth displayed disorganized hair bundles, including displaced kinocilia, resulting in distorted stereocilia development.

## 5. Mutant Mice for Genes Responsible for Endolymph Production

The cochlea is an interesting organ with regards to electrophysiology. The perilymphatic space contains a fluid (perilymph) with a high Na^+^ and low K^+^ concentration. The apices of the hair cell are facing the endolymph, which is just the opposite in its ionic composition (low Na^+^, high K^+^), but its basal surface is immersed in perilymph. Movement of K^+^ in the cochlea from perilymph to endolymph through the cochlear lateral wall and maintenance of the ionic composition of the endolymph are critical for auditory function. Mutant mice for genes involved in K^+^ movement and recycling have been instrumental in the understanding of this critical process.

 There are several genes known to account for HLL in human which have a role to play in K^+^ recycling, and mutant mouse were developed for the some of them which include *Gjb2/Cx26, Gjb6/Cx30, *and *Cldn14 *that encode intercellular adhesion proteins. *Gjb2 *and *Gjb6 *genes produce the gap junction proteins, connexin 26 (Cx26) and connexin 30 (Cx30), while *Cldn14* codes for a tight-junction protein; *Kcne1, Kcnq1, *and *Kcnq4, *all encoding for potassium ion channels; and finally *Slc26a4 *that encodes an anion transporter.

In humans, *GJB2 *and *GJB6 *genes are located in the same chromosomal locus (13q11-12). Mutations in this locus account for a very high incidence of congenital hereditary NSHL with slight variability over populations. The causative mutations are small deletions in the *GJB2 *gene and they are autosomal recessive in nature. Over a hundred deafness-related different mutations in *GJB2 *have been identified in humans so far.

Both targeted mutagenesis [43] and chemical (ENU-induced) mutagenesis [44] have been used to knock out the *Gjb2/Cx26 *gene in mice. Both of these methods resulted in an identical phenotype. While the heterozygotes had no auditory defects, the homozygotes had prenatal death due to placental defects in the mother. The absence of any auditory phenotype led to the employment of two additional strategies to generate hearing-impaired viable mutant *Gjb2 *mouse models. *Gjb2 *was conditionally knocked out in the cochlear epithelial network, using the *cre-loxP *system by crossing the *Gjb2-loxP *mouse to a mouse carrying the *Cre *under the *Otog *promoter, which is expressed only in cochlear epithelial cells [18]. In the other approach, point mutagenesis was employed to create the *Cx26^R75W^* mutation [45] that is causative of autosomal dominant SHL (HHL and skin disease) in human heterozygotes [46]. The dominant inheritance was attributed to the ability of the mutant Cx26 to inhibit the function of gap junctions that coassemble wild-type and mutant Cx26 molecules.

Both homozygotes for *Gjb2* conditional knockout and heterozygotes *Cx26^R75W^* exhibited similar auditory defects in adults and histological profiles, although the point mutant displayed a much more severe phenotype. In both mice, the inner ear developed normally until postnatal day 14 (P14). Only after the onset of auditory function, at P15-P16, epithelial cells began to die mostly due to apoptosis. The IHC-neighboring supporting cells were first damaged, followed by OHC and their supporting cells. The tunnel of Corti was collapsed. Heterozygotes for* Cx26^R75W^* displayed degeneration of all organs of Corti that began at P14 and led to a complete degradation of both hair cells and supporting cells by seven weeks after birth. It is interesting to note that in *Gjb2 *knockout mice, IHC died only in the severely hearing-impaired mice, and some of the intradental cells of the spiral limbus were degenerated at a much older age (P60). The reticular lamina at the apical surface of the sensory epithelium was disrupted from an early stage in *Gjb2 *knockout mice. Therefore, it appears that, though Cx26 is not required for the normal development of the organ of Corti, it is essential for its survival and function.

Following reports of recessive mutations of human *CLDN14 *as causative of extreme NSHL in humans [47], *Cldn14*-null mutant mice were created to explore the role this gene plays in the inner ear function and morphogenesis. *Cldn14*-null mice had a normal EP, but were deaf. There were no discernible vestibular phenotypes. Although the reticular lamina tight junctions seemed normal microscopically in null mice, the hair cell stereocilia were lost or disintegrated during the first 3 weeks of life, rapidly followed by hair cell degeneration. Unlike the Cx26 mutant, here OHCs were degenerated before IHC.

The genes *Kcne1, Kcnq1, *and *Kcnq4 *encode for subunits of slow voltage activated potassium channels, which are critical components of cellular repolarization in excitable cells. They open during depolarization and facilitate selective efflux of K^+^ across semipermeable membranes. *Kcne1* [48] or *Kcnq1* [49, 50] knockout mice exhibited a classic waltzer-like phenotype with severe hearing loss and vestibular symptoms, leading to complete deafness in adult mice. Although these mice display normal anatomical structure of the inner ear at birth, there are rapid changes later. The strial marginal cells and the vestibular dark cells were unable to secrete K^+^ ions, leading to degeneration of the neuroepithelium including the hair cells and complete collapse of the endolymphatic space.

M type channels are very slow-voltage-dependent K^+^ channels, and Kcnq4 is an alpha subunit of such a channel. In humans, *KCNQ4 *mutations induce autosomal dominant NSHL, suggesting that the mutated gene has a dominant negative effect when it is coexpressed with the wild-type allele [51]. Two mutant mouse models for *Kcnq4 *are available: a homozygous knockout mouse and a mouse with a point mutation that mimics the dominant negative mutation in humans. Surprisingly, no vestibular phenotypes were observed in both these mutants, although Kcnq4 is strongly expressed in vestibular hair cells. The mice had normal hearing at and after birth, but displayed a progressive hearing impairment that was accompanied with a degeneration of OHC. The progression of both deafness and OHC loss was much faster in homozygotes (both for knockout as well as point mutant) over the heterozygotes. In the homozygotes the phenotype was detected within weeks as compared to months in heterozygotes. A selective inhibitor of Kcnq channels was used to isolate Kcnq-dependent K^+^ currents, and both from the OHC homozygous, or dominant negative heterozygous Kncq-dependent K^+^ currents were detected. The absence of the K^+^ current led to depolarized resting membrane potentials of the OHC. IHCs were not significantly affected. Therefore, it was proposed that *Kcnq4 *mutations induce a progressive HHL due to chronic depolarization of OHC, leading to their degeneration [52].

The transportation of several anions, including chloride, iodide, sulfate, nitrate, bicarbonate, hydroxyl, oxalate, and formate constitutes the main function of the SLC26 (solute carrier protein 26) family of anion exchangers. These are integral proteins with 10–12 transmembrane domains. Each member in this family has different affinity and specificity for different anions. Two members of the SLC26 have been associated with HHL in humans: SLC26A4/pendrin and SLC26A5/prestin. *SLC26A4* mutations were associated with both SHL (Pendred syndrome) [53] and NSHL [54], while *SLC26A5 *was associated only with NSHL [55].

*Slc26a4 *knockout mice (*Pds*−*/*−) exhibited waltzer-like vestibular dysfunction and complete deafness. Their inner ears developed normally only until E15, which is two days after the start of pendrin expression in wild-type mice. Thereafter, endolymphatic cavities were severely dilated, both in cochlea and vestibule. This dilatation was proposed to be secondary effect due to a changed osmotic condition and a much increased volume of the endolymphatic fluid. During the second postnatal week, hair cells began to degenerate. In the vestibule, the otoconia and otoconial membranes were also destructed [56]. After weaning, the strial vascularis marginal cells of *Pds*−*/*− mice displayed irregular shapes and sizes, resulting in a thinner stria vascularis.

## 6. Mutant Mice for Extracellular Matrix Components (ECM)

The basilar membrane, composed of collagen, is the connective tissue on which sits the cochlea harboring the organ of corti. The basilar membrane is of various stiffness along the cochlea and resonates due to sound-induced movements of the cochlear fluids. These vibrations are detected by two types of hair cells, included in the sensory epithelium of the organ of Corti and the inner and outer hair cells (IHC and OHC), respectively. Though so far seven known collagens have been linked with Human HLL: COL2A1, COL4A3, COL4A4, COL4A5, COL9A1, COL11A1, and COL11A2, only five of these have a mutant mouse model (*Col11a1, Col11a2, Col2a1, Col4a3, Col9a1*). A *COL11A2 *mutation was linked to autosomal dominant NSHL and Stickler syndrome in human. The other collagen genes were only associated with SHL in humans, mainly Stickler (*COL2A1*,* COL9A1 *and *COL11A1*) and Alport (*COL4A3-5*) syndromes. Because the human *COL4A3 *gene is causative for Alport syndrome [57], a mouse knockout for *Col4a3 *was generated [58]. Homozygotes died at about 14 weeks of age due to renal failure. Postmortem analysis revealed that renal glomeruli had defective basement membranes and cochlear membranous labyrinth was highly degenerated, mimicking the human condition. Both Col4a3 and Col4a4 were found to be completely absent in the cochlear labyrinth. Basement membranes of different regions of the membranous labyrinth were significantly different in thickness or were completely absent when compared to wild type cochleae, and there were collapsed capillaries nearby. Renal and cochlear defects were highly progressive and HI was detected only after 6 weeks.

In addition to sensorineural HHL, Stickler syndrome also causes degenerative changes in various joints with abnormal bone development, vertebral abnormalities, osteoarthritis, and in severe cases unusual cleft palate. The Stickler syndrome-related collagens, Col2a1, Col11a1, and Col11a2, are important components of both the cochlear TM and cartilage. COL2A1 was found to be involved in sensorineural deafness that is associated with hereditary syndromes in humans, like Stickler syndrome, spondyloepiphyseal dysplasia congenita (SEDC), and chondrodysplasia. A *Col2a1* mutant mouse was generated by irradiation in 1966. This mutant had a three-nucleotide deletion in the region encoding the C-propeptide globular domain of Col2a1 recapitulating the Dmm condition in humans. *Dmm *mice expressed a reduced level of collagen II and suffered from cartilage defects that affect inner ear development as well [59].

The *cho *mutation is a single nucleotide deletion in the *Col11a1 *gene that causes a frameshift and a premature stop codon. *Cho *mice are spontaneously arisen mutant mice [60]. Homozygotes had a cleft palate and had postnatal death due to lethal chondrodysplasia. The premature termination of translation leads to an incomplete protein that is incapable of assembling with other collagen molecules. Homozygotes were severely hearing-impaired at birth due to underdevelopment of the organ of Corti in the lower turn of the cochlea, with no hair cells, supporting cells, nerve endings, and pillar cells [61].

 A *Col11a2 *knockout mouse was generated by insertional mutagenesis by inserting a neomycin resistance cassette in exons 27 and 28 of the gene. The inserted sequence included a premature termination codon, resulting in a truncated protein product. The phenotype was much less severe compared to *cho *mice. The only observable phenotype in the inner ear was a larger and less compact TM with disintegrated collagen fibrils [62, 63].

## 7. Micro-RNAs in Deafness

In recent years, there is an increasing focus on noncoding RNAs and their contribution to regulation of normal development as well as disease. It is now established that the miR-183 family of miRNAs is expressed specifically in the innerear hair cells and the eye retina in mammals [64, 65], and other studies have reported that at least 100 different miRNAs are present in the developing mouse inner ear [66]. Recently, two groups have located point mutations in the seed region of the *miR-96* with autosomal dominant progressive NSHL in humans [67] and mice [68]. In human, two different mutations (transversion and transition) in neighboring nucleotides (13G > A and 14C > A) of the MIR96 gene were observed in two unrelated Spanish families. The hearing loss in the two families was not identical. These mutations significantly affected the biogenesis of mature miR-96 or increased its degradation. In addition, these mutations changed the target mRNA population which was targeted by the miRNA, and the mutated miR-96 had an impairment in its ability to downregulate the translation of several mRNAs that are targeted by wild-type miR-96. Other substitutions in the pre-miRNA sequences of MIR96 and MIR182 genes, outside the mature miRNA sequences, were found in 10 and 22 families [67], respectively, but did not segregate with the hearing loss phenotype. Although from the mouse study the link between the direct targets of the miRNA and the phenotype are not clear, several genes known to be important for hair cell function are specifically downregulated in the diminuendo mutant and any one could account for the hair cell dysfunction.

## 8. Conclusion

The sense of hearing is one of the most crucial senses endowed to a living organism and its loss can have many ramifications. Finding the causes, both genetic and environmental, goes a long way in understanding this common disability. The mouse has been an invaluable ally in our understanding of the various genetic components which underlie hearing impairment. Efforts are underway to knock out all the genes in the mouse genome and this will undoubtedly give rise to many more models for HLL. The future requires research in complex hearing impairment and a study of complex/multiple mutants to obtain a better molecular handle of the various genetic interactions occurring in both the normal, as well as diseased ear. There is also a growing need to look at this problem from a systems biology angle by simultaneously deciphering the multiple genes and their regulatory networks involved in hearing loss and to capture the earliest set of genes involved in the auditory processes and their downstream targets. With the knowledge accumulated, it will eventually make hearing impairment easier to detect, manage and rectify at the genetic level.
